# Decorin expression is associated with predictive diffusion MR phenotypes of anti-VEGF efficacy in glioblastoma

**DOI:** 10.1038/s41598-020-71799-w

**Published:** 2020-09-09

**Authors:** Kunal S. Patel, Jingwen Yao, Catalina Raymond, William Yong, Richard Everson, Linda M. Liau, David Nathanson, Harley Kornblum, Chencai Wang, Talia Oughourlian, Albert Lai, Phioanh L. Nghiemphu, Whitney B. Pope, Timothy F. Cloughesy, Benjamin M. Ellingson

**Affiliations:** 1 UCLA Brain Tumor Imaging Laboratory (BTIL), Center for Computer Vision and Imaging Biomarkers (CVIB), Dept. of Radiological Sciences, David Geffen School of Medicine, University of California Los Angeles, 924 Westwood Blvd, Suite 615, Los Angeles, CA 90024 USA; 2 Dept. of Neurosurgery, David Geffen School of Medicine, University of California Los Angeles, Los Angeles, CA USA; 3 Dept. of Radiological Sciences, David Geffen School of Medicine, University of California Los Angeles, Los Angeles, CA USA; 4 UCLA Neuro-Oncology Program, David Geffen School of Medicine, University of California Los Angeles, Los Angeles, CA USA; 5 Dept. of Pathology, David Geffen School of Medicine, University of California Los Angeles, Los Angeles, CA USA; 6 Dept. of Pharmacology, David Geffen School of Medicine, University of California Los Angeles, Los Angeles, CA USA; 7 Dept. of Psychiatry and Biobehavioral Sciences, David Geffen School of Medicine, University of California Los Angeles, Los Angeles, CA USA; 8 Neuroscience Interdisciplinary Program, David Geffen School of Medicine, University of California Los Angeles, Los Angeles, CA USA; 9 Dept. of Neurology, David Geffen School of Medicine, University of California Los Angeles, Los Angeles, CA USA

**Keywords:** Cancer imaging, CNS cancer

## Abstract

Previous data suggest that apparent diffusion coefficient (ADC) imaging phenotypes predict survival response to anti-VEGF monotherapy in glioblastoma. However, the mechanism by which imaging may predict clinical response is unknown. We hypothesize that decorin (DCN), a proteoglycan implicated in the modulation of the extracellular microenvironment and sequestration of pro-angiogenic signaling, may connect ADC phenotypes to survival benefit to anti-VEGF therapy. Patients undergoing resection for glioblastoma as well as patients included in The Cancer Genome Atlas (TCGA) and IVY Glioblastoma Atlas Project (IVY GAP) databases had pre-operative imaging analyzed to calculate pre-operative ADC_L_ values, the average ADC in the lower distribution using a double Gaussian mixed model. ADC_L_ values were correlated to available RNA expression from these databases as well as from RNA sequencing from patient derived mouse orthotopic xenograft samples. Targeted biopsies were selected based on ADC values and prospectively collected during resection. Surgical specimens were used to evaluate for DCN RNA and protein expression by ADC value. The IVY Glioblastoma Atlas Project Database was used to evaluate DCN localization and relationship with VEGF pathway via in situ hybridization maps and RNA sequencing data. In a cohort of 35 patients with pre-operative ADC imaging and surgical specimens, DCN RNA expression levels were significantly larger in high ADC_L_ tumors (41.6 vs. 1.5; P = 0.0081). In a cohort of 17 patients with prospectively targeted biopsies there was a positive linear correlation between ADC_L_ levels and DCN protein expression between tumors (Pearson R^2^ = 0.3977; P = 0.0066) and when evaluating different targets within the same tumor (Pearson R^2^ = 0.3068; P = 0.0139). In situ hybridization data localized DCN expression to areas of microvascular proliferation and immunohistochemical studies localized DCN protein expression to the tunica adventitia of blood vessels within the tumor. DCN expression positively correlated with VEGFR1 & 2 expression and localized to similar areas of tumor. Increased ADC_L_ on diffusion MR imaging is associated with high DCN expression as well as increased survival with anti-VEGF therapy in glioblastoma. DCN may play an important role linking the imaging features on diffusion MR and anti-VEGF treatment efficacy. DCN may serve as a target for further investigation and modulation of anti-angiogenic therapy in GBM.

## Introduction

Every year in the United States, approximately 44,000 new primary brain tumors are diagnosed^1^. Of these newly diagnosed tumors, approximately 60% are malignant and 45% are gliomas^1^. Glioblastoma (GBM) is a particular type of infiltrative malignant glioma that is trademarked by a very poor patient prognosis, with median overall survival (OS) of around 14 months^2^ and less than a 10% survival rate at 5 years^3^. Despite relatively ineffective therapies and a 100% fatality rate, a few strong prognostic and predictive biomarkers exist that may provide some therapeutic guidance based on slight survival benefits for patients with GBM. For example, O-6-methylguanine-DNA methyltransferase (MGMT) promoter methylated patients have been shown to significantly benefit from temozolomide or other alkylating agents^4^, IDH mutant GBMs are highly responsive to radiation therapy^5–7^ and have significantly longer overall survival irrespective of therapy or number of recurrences^8–15^. Interestingly, pre-treatment diffusion-weighted magnetic resonance imaging (DWI or DW-MRI) estimates of the apparent diffusion coefficient (ADC) within the enhancing lesion have been shown to be *predictive* for survival benefit on anti-VEGF treatment in the recurrent setting in both single-center^16–18^ and multicenter studies^19–21^ suggesting a potential mechanistic link between water mobility within the tumor and anti-VEGF treatment efficacy.

Despite these observations, only a few, rather simplistic biologic associations based on changes in cell structure and density have been identified and associated with changes in measured ADC in the central nervous system. For example, increased ADC in the brain and spinal cord has been associated with decreased axon^22^ or dendrite density and myelin sheath thickness^23,24^, as these represent relatively impermeable barriers and restrictions to water diffusion. Similarly, a decreased ADC in brain tumors had been associated with increased cell density^25–30^ and mitotic index^31^. However, these strictly “structural” associations do not explain the many discrepancies reported in the post-therapeutic setting or why diffusion MR phenotypes would be particularly predictive in anti-VEGF treatment. Thus, the mechanism or rationale for how pre-treatment ADC measurements predict response to anti-VEGF therapy in glioblastoma remains unclear.

A differential gene expression study from our laboratory identified overexpression of decorin (DCN) as a possible mechanism for altered water diffusivity^32^ and anti-VEGF efficacy. Specifically, DCN may increase water diffusivity through direct modulation (softening) of the extracellular matrix (ECM) along with decreased tumor cell proliferation. DCN acts to modulate the rigidity and stiffness of the ECM by binding with various ECM macromolecules and activating specific matrix metalloproteinases (MMPs)^33^. DCN injections soften fibrotic connective and scar tissues within body tissues^34–41^ and the central nervous system (i.e. gliotic scaring)^42–44^, suggesting DCN may increase fluid mobility within the extracellular environment through remodeling of the ECM. This is further evidenced by studies showing that DCN expressing viral vectors that transfect tumor tissues can improve diffusion and penetration of macromolecules including chemotherapies^45–47^. The protein core of DCN binds with a variety of collagen molecules, fibrils, and other macromolecules, acting to significantly increase inter-fibrillary spacing^48^. Since ADC is inversely correlated with fluid viscosity^49,50^ and tortuosity of the ECM^51–55^, it is conceivable that high DCN expression results in softer and less viscous tumors with decreased boundaries to the fluid within the extracellular space from DCM remodeling, ultimately resulting in a higher measured ADC.

In addition to softening the ECM, DCN also reduces the inherent growth rate of malignant tumors. DCN acts as a tumor repressor gene^56–58^, suppresses growth by evoking expression of p21^59^, and causes prolonged suppression of cellular signaling required for cell survival and proliferation via different RTKs^60^. DCN-null mice develop spontaneous tumors^61^ and have increased tumor growth rates^62^. An anti-oncologic role for DCN has been documented in breast cancer^63^, ovarian carcinoma^64^, rat gliomas^65^ and squamous and colon carcinoma xenografts^66–68^. Attenuated DCN expression is associated with poor prognosis in invasive breast cancer^69^, soft tissue tumors^70^, mammary gland carcinogenesis^71^, and GBM. Additionally, ectopic expression of or exogenous treatment with DCN inhibits tumor growth in a variety of preclinical models^59,60,65,72–74^.

An abundance of scientific data (reviewed in^33,74–76^) implicates DCN a multifaceted anti-angiogenic agent in a variety of malignancies^33,77–80^ with DCN expression inversely proportional to extent of vascularity^81^. DCN interferes with thrombospondin-1^77^ and suppresses endogenous tumor cell production of VEGF family proteins^33,78^ including VEGF-A^82^, the therapeutic targets of bevacizumab and other anti-VEGF therapies. DCN also acts to inhibit VEGF receptor 1 and 2 (VEGFR1/2) activation by binding with a high affinity^83^. Resistance to bevacizumab has been shown to result in high expression of VEGFR1 and high concentrations of soluble, circulating VEGFR1 in a variety of tumor types^84–88^. Thus, DCN within the tumor environment may act to inhibit escape through VEGFR-specific mechanisms. Additionally, stromal DCN directly abrogates the HGF/Met signaling axis and inhibits VEGF-mediated angiogenesis by transcriptionally repressing HIF-1α, β-catenin, Myc, and SP1^89^. DCN also acts as an anti-angiogenic factor by binding directly to a variety of additional cell surface receptors or signaling molecules involved in angiogenesis. For example, DCN is known to be a ligand to EGFR^90,91^ and Met, the receptor for hepatocyte growth factor (HGF)^92^, insulin-like growth factor receptor-I (IGF-IR)^93–95^, and with platelet derived growth factor receptor (PDGFR)^96^. DCN influences the expression and bioavailability of several angiogenic growth factors and cytokines including downregulation of monocyte chemoattractant protein-1 (MCP-1) and angiopoietin^97^. DCN also interacts with a variety of other proangiogenic factors including platelet derived growth factor (PDGF)^96,98,99^, fibroblast growth factor (FGF)^100,101^, IGF^93–95^, connective tissue growth factor (CTGF)^102–104^, and HGF^92,105^. Additionally, DCN overexpression directly inhibits transforming growth factor beta (TGF-β) expression^38^ and the DCN molecule itself binds directly to TGF-β with a high affinity^106–109^. TGF-β is a strong angiogenic factor^110–114^ that is greatly overexpressed in GBM cell lines driven to resistance to bevacizumab^115^, further supporting the hypothesis that DCN expression and presence in the extracellular environment may increase survival in patients with recurrent GBM through prolonged resistance to bevacizumab by obstructing pro-angiogenic escape pathways.

In the current study, we explore the association between DCN gene expression and diffusion MRI measurements in two large independent cohorts of GBM patients from The Cancer Genome Atlas (TCGA) and IVY Glioblastoma Atlas Project (IVY GAP). We correlate pre-operative diffusion MRI measurements to DCN RNA expression using bulk RNA sequencing from patient derived xenograft models and DCN protein expression using immunohistochemistry (IHC). Using available in situ hybridization and RNA sequencing data from the IVY GAP database and IHC from clinical samples we illustrate expression of DCN in areas corresponding to microvascular proliferation on histology. We report an association of diffusion phenotypes to overall survival in a cohort of patients treated with bevacizumab as well as a similar survival benefit in patients with elevated DCN expression. We begin to investigate the specific mechanism of DCN, finding a positive correlation of expression and co-localization of DCN with VEGFR-1 & 2. Our data suggest that DCN serves as a biomarker for underlying GBM than can be monitored using diffusion MRI and a candidate for modulation of anti-angiogenic activity.

## Results

### Elevated ADC_L_ is associated with increased DCN mRNA expression

**Figure 1:**
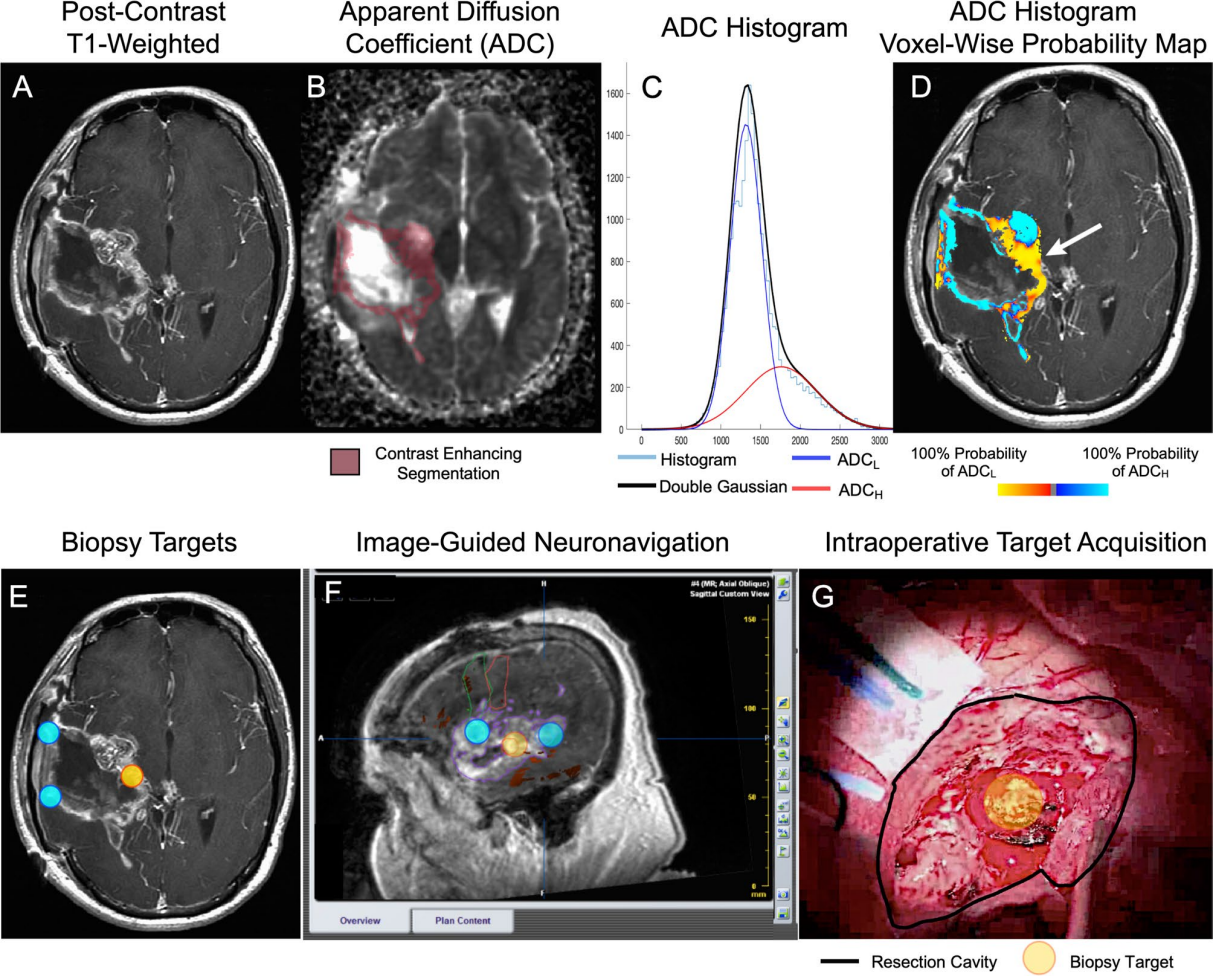
ADC histogram analysis and targeted biopsy acquisition. Pre and post contrast T1 MR images were acquired (**A**) to evaluate ADC levels of each voxel of enhancing tumor (**B**) to yield an ADC histogram (**C**). A double gaussian model (black line) was applied to this histogram (light blue line) to identify the higher (ADC_H_, red line) and lower (ADC_L_, blue line) distributions. A probability map (**D**), representing probability of a voxel occurring in the ADC_L_ distribution was used to prospectively identify biopsy targets (**E**). These targets were loaded onto T1 with contrast images on intraoperative neuronavigation software (**F**) to facilitate tissue collection during tumor resection (**G**).

**Figure 2:**
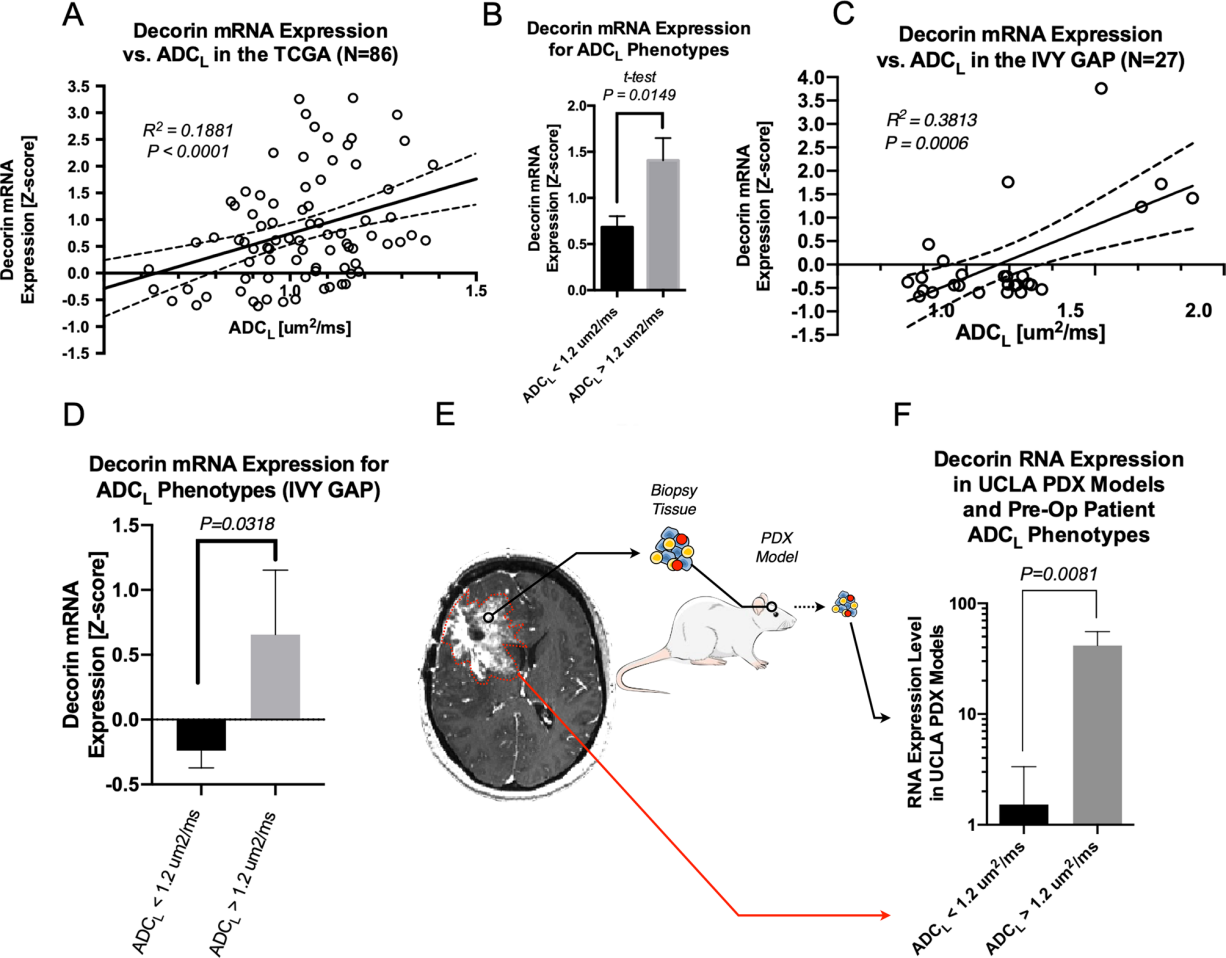
Diffusion imaging phenotypes correlate with DCN RNA expression. Data from the TCGA and TCIA databases showed a positive correlation between ADC_L_ and Z-score normalized RNA expression levels of DCN (**A**) and higher expression in patients with high ADC_L_ (**B**). Similarly, data from the IVY GAP database showed a positive correlation between ADC_L_ and Z-score normalized RNA expression levels of DCN (**C**) and higher expression in patients with high ADC_L_ (**D**). RNA expression levels from PDOX models (**E**) when derived from patients with high and low preoperative ADC_L_ (**F**).

Previous data from our laboratory have shown that there is differential gene expression in GBM exhibiting high versus low ADC_L_^32^. In this experiment, DCN overexpression was identified as a key differentially expressed gene. To evaluate this further, we carried out ADC histogram analysis (*Methods*) on pre-treatment MRI scans (Fig. 1A-D) and obtained prospective biopsy specimens from areas of different ADC (Fig. 1E-G). We identified 86 patients with RNA expression data and suitable MR imaging using the TCGA/TCIA databases. There was a positive linear correlation between DCN RNA expression Z-score and ADC_L_ (Pearson R^2^ = 0.1881; P < 0.001; Fig. 2A). When these data were stratified by high and low ADC_L_ using previously defined, optimized thresholds (ADC_L_ > 1.24 µm^2^/ms), DCN expression was significantly higher in the elevated ADC_L_ group (P = 0.0149; Fig. 2B). Next, we identified 27 patients with RNA expression data and suitable MR imaging using the IVY GAP database. Similarly, we observed a positive linear correlation between DCN RNA expression Z-score and ADC_L_ (Pearson R^2^ = 0.3813; P = 0.0006; Fig. 2C). When these data were stratified by high and low ADC_L_, DCN expression was significantly higher in the elevated ADC_L_ group (P = 0.0318; Fig. 2D).

We confirmed these data with a clinical cohort at our institution. A group of 35 patients undergoing resection of new or recurrent GBM underwent pre-operative ADC histogram analysis and after resection, tumor specimen was used to establish patient derived mouse orthotopic xenograft (PDOX) models (Fig. 2E). Purified tumor tissue from patient derived mouse xenograft models were harvested and evaluated for DCN RNA expression levels and compared to preoperative ADC histogram analysis results performed in the human patients from which the PDOX tumors were derived. Similar to the TCGA and IVY GAP datasets, DCN RNA expression levels from PDOX models were significantly higher in tumors with elevated ADC_L_ in human patients prior to surgery (41.6 vs. 1.5; P = 0.0081; Fig. 2F).

### Increased ADC_L_ is associated with increased intertumoral and intratumoral DCN protein expression

**Figure 3:**
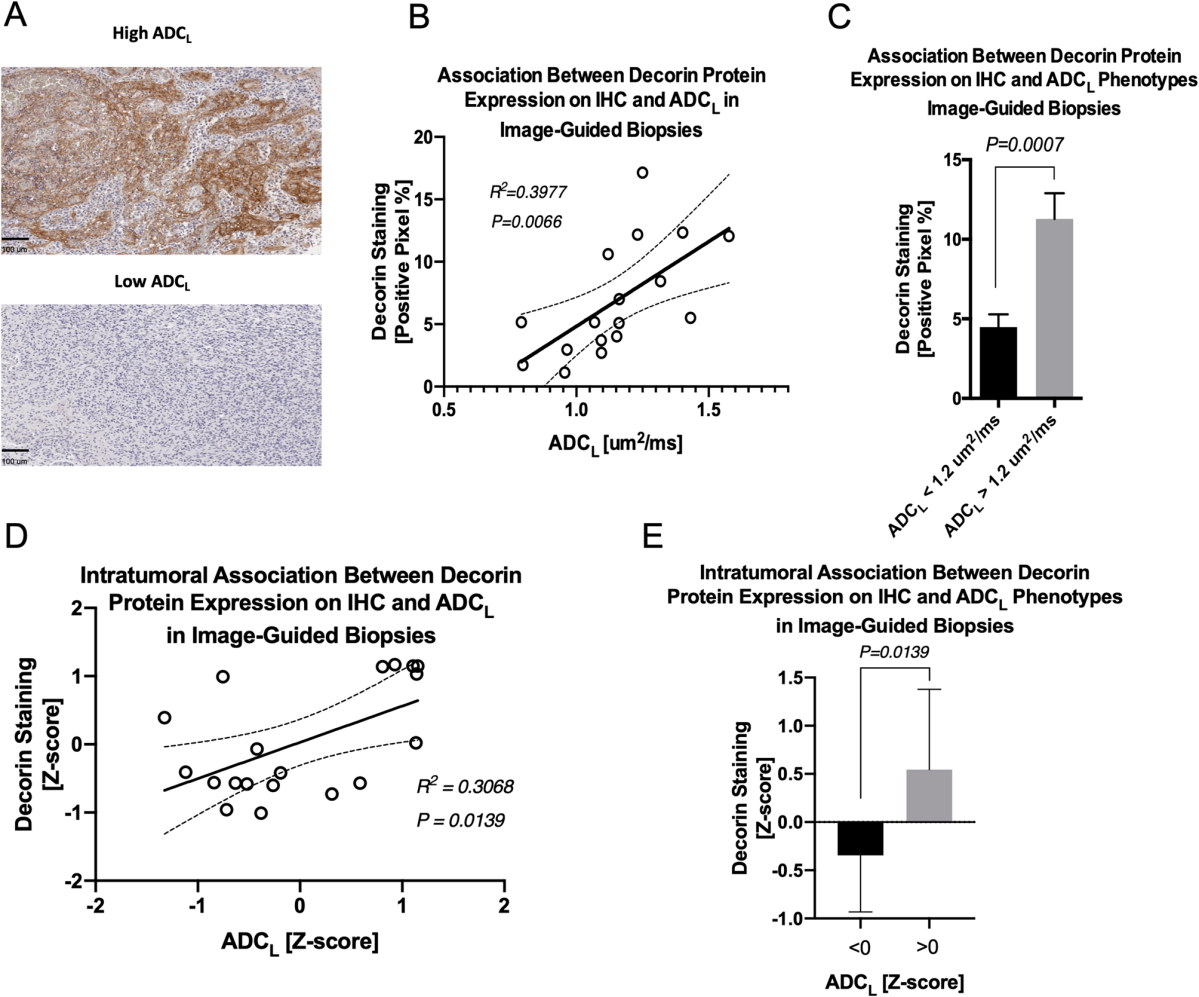
Diffusion imaging phenotypes correlate with DCN protein expression. Patients with targeted biopsy specimens were stained for anti-DCN antibodies (**A**). There was a positive correlation between percent positive pixel staining on IHC and ADC_L_ from preoperative ADC probability maps (**B**) and higher staining in patients with high ADC_L_ (**C**). Samples with multiple intratumoral biopsies were Z-score normalized and demonstrated a positive correlation between DCN staining and ADC_L_ (**D**) and staining was higher in patients with high ADC_L_ (**E**).

A group of 17 patients undergoing resection of new or recurrent GBM were prospectively identified to undergo pre-operative ADC histogram analysis. Tumor tissue was stained for DCN in each patient and positive pixel percentage was calculated (Fig. 3A). There was a positive correlation between ADC_L_ and DCN staining on IHC (Pearson R^2^ = 0.3977; P = 0.0066; Fig. 3B). When these data were stratified by high and low ADC_L_, DCN protein expression was significantly higher in the high ADC_L_ group (11.3% vs. 4.5%; P = 0.0007; Fig. 3C). In 6 patients undergoing resection of new or recurrent GBM, multiple samples of tumor tissue were obtained corresponding to areas of high and low ADC within the same tumor. There was a positive correlation between normalized ADC_L_ Z-scores and normalized DCN staining Z-scores (Pearson R^2^ = 0.3068; P = 0.0139; Fig. 3D). When these data were stratified by high and low ADC based on previously optimized thresholds, DCN protein expression was significantly higher in the high ADC_L_ group (0.545 vs. − 0.346; P = 0.0139; Fig. 3E).

### DCN is localized to blood vessels in GBM

**Figure 4:**
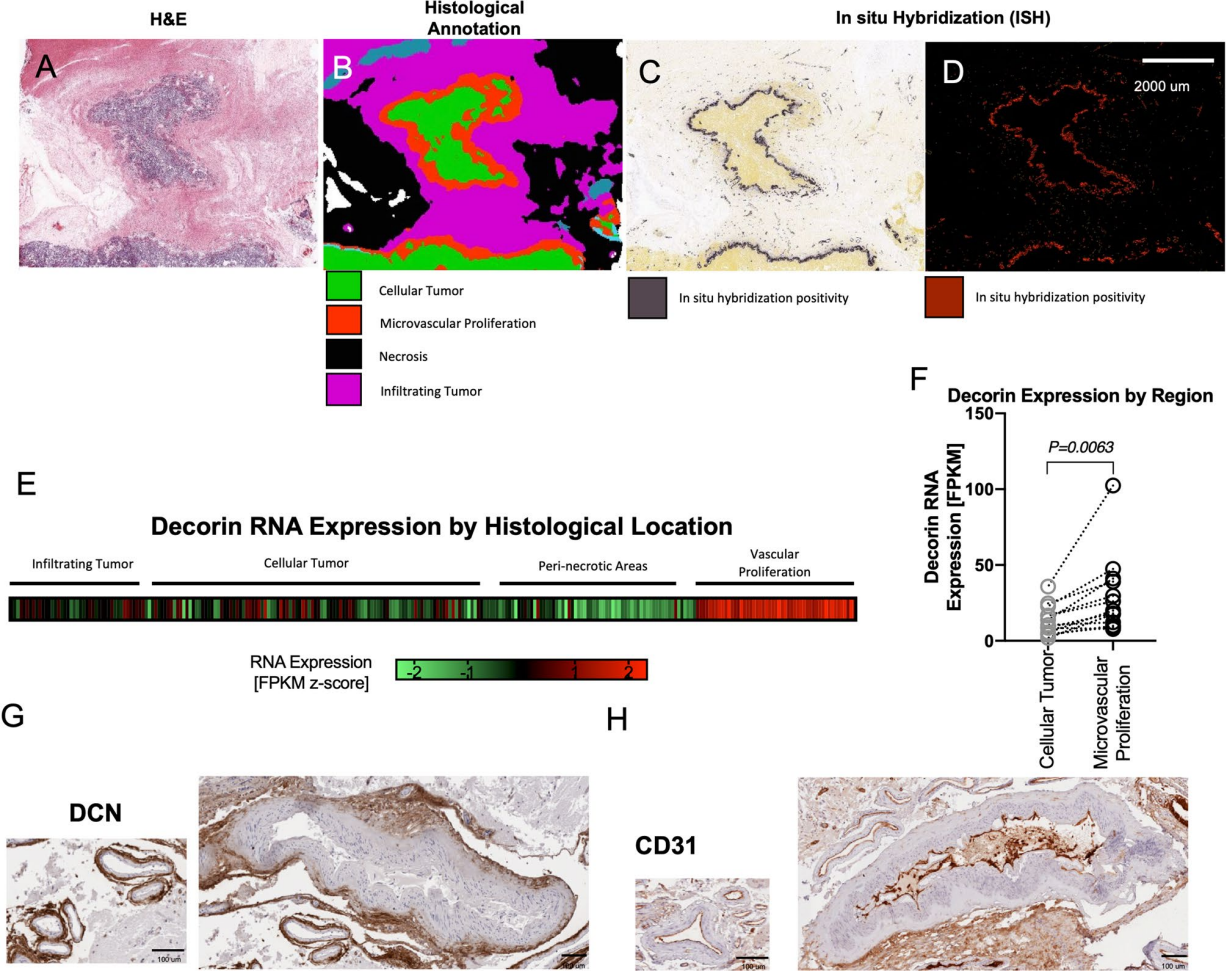
DCN expression localizes to tunica adventitia of areas of vascular proliferation in GBM. Pathological images downloaded from the IVY GAP database including H&E (**A**), histological annotation map (**B**), and ISH (**C**,**D**) illustrated localization of DCN to areas of microvascular proliferation. Normalized RNA sequencing data from the IVY GAP database showed elevated expression in areas of microvascular proliferation (**E**). Samples with RNA expression data from different histological areas of tumor showed increased expression in areas of microvascular proliferation in a paired analysis (**F**). Comparisons of IHC staining of DCN (**G**) and CD31 (**H**) showed localization of DCN to tunica adventitia of vessels.

To investigate the role and regional localization of DCN in GBM, we queried the IVY GAP in situ* hybridization* (ISH) database to evaluate localization of DCN RNA expression. Specifically, we reviewed H&E sections of clinical GBM specimens (Fig. 4A), histological annotation maps of associated sections (Fig. 4B), and ISH positivity maps of associated sections (Fig. 4C,D) to identify DCN ISH positivity localized to areas of microvascular proliferation. This finding was corroborated by analysis of RNA sequencing data from 271 tumor specimens associated with different histological locations from 37 different tumors downloaded from the database. DCN RNA expression levels were significantly higher in demarcated areas of microvascular proliferation than areas of cellular tumor, infiltrating tumor, or perinecrotic regions (Fig. 4E). In 13 cases, there were DCN RNA expression levels for both regions of cellular tumor and microvascular proliferation. In this subset, DCN RNA expression was significantly elevated in microvascular proliferation relative to cellular areas from the same tumor (Paired t-test; P = 0.0063; Fig. 4F).

In image-guided biopsy patients, sections stained for DCN and CD31 were used to localize DCN protein expression. Consistently, DCN expression was localized to the tunica adventitia of blood vessels (Fig. 4G) while CD31 localized, as expected, to the tunica intima (Fig. 4H).

### Association of DCN with VEGF pathway

**Figure 5:**
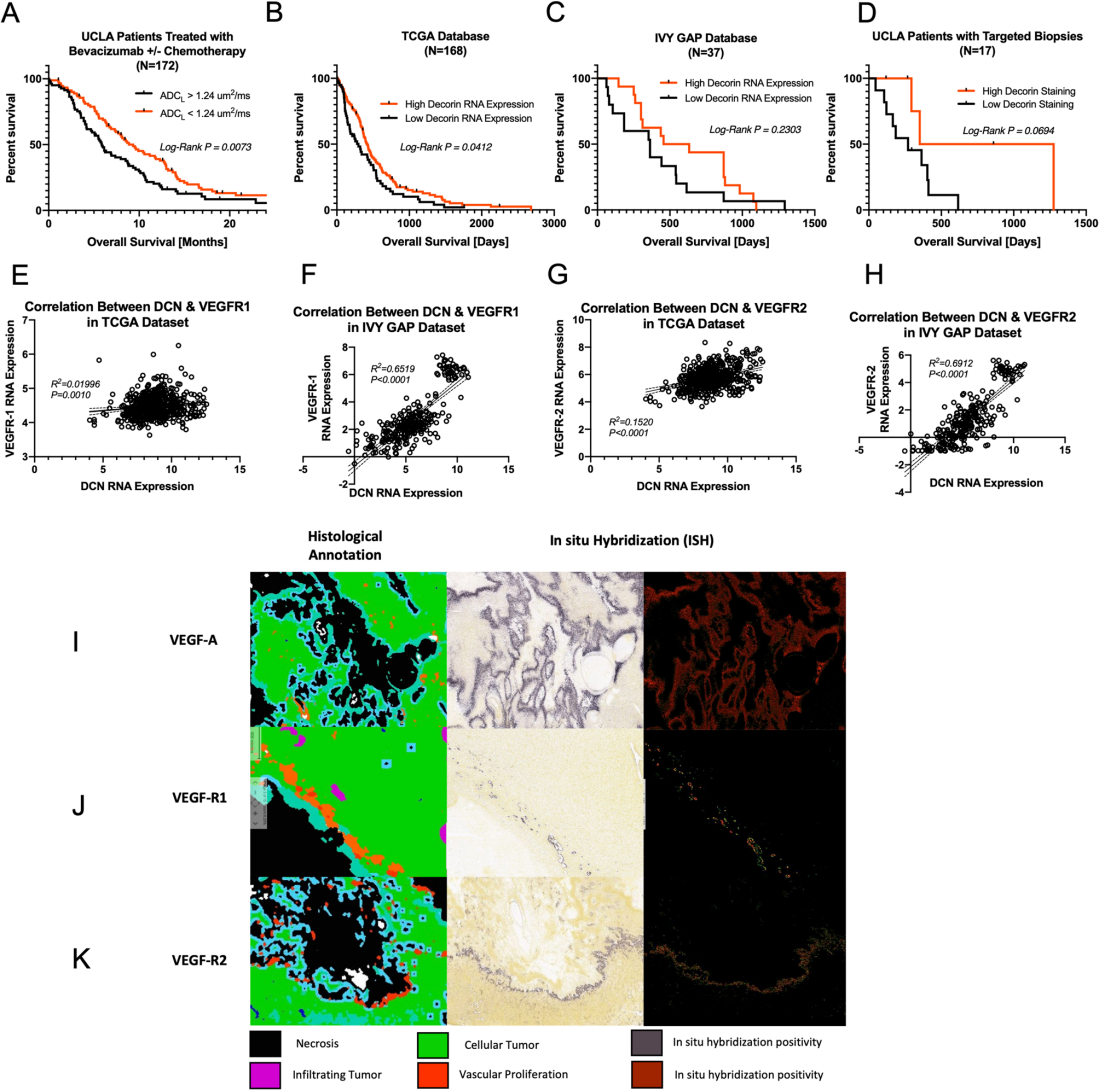
DCN is associated with increased survival and co-localizes with VEGFR 1/2. Increased ADC_L_ was associated with increased survival in a series of UCLA patients treated with bevacizumab (**A**). Increased DCN expression was associated with increased survival using data from the TCGA (**B**), IVY GAP (**C**) and UCLA patients (**D**). There was a positive correlation between DCN and VEGF R1 in the TCGA (**E**) and IVY GAP (**F**) as well as VEGF R2 in the TCGA (**G**) and IVY GAP (**H**). Using ISH and histological annotations from IVY GAP, VEGFA was found to be associated in areas of cellular tumor (**I**) and VEGFR1/2 in areas of microvascular proliferation (**J**,**K**).

We have previously found that ADC phenotypes are associated with differences in overall survival when treated with VEGF inhibitors in data from large clinical trials^16–21,116^. To confirm these previous observations, we retrospectively reviewed electronic medical record charts to identify survival data for a cohort of 172 patients treated bevacizumab with or without chemotherapy for recurrent glioblastoma. Consistent with previous findings, patients with high ADC_L_ had significantly longer overall survival relative to patients with low ADC_L_ (Median Survival 9.33 vs. 5.87; Log-Rank P = 0.0073; Fig. 5A).

Given the findings that (1) higher ADC is associated with increased overall survival in patients treated with anti-VEGF therapy and (2) higher ADC is associated with higher levels of DCN expression we investigated whether DCN expression levels themselves were associated with differences in overall survival. We gathered survival and RNA expression data from the TCGA and IVY GAP databases and stratified by median DCN expression. In the TCGA database (n = 168) there was a significant increase in overall survival with high DCN expression (Median Survival 13.6 vs. 9.8 months; P = 0.0412; Fig. 5B). In the IVY GAP database (n = 37) there was a trend towards increased overall survival with high DCN expression (Median Survival 17.9 vs. 11.9 months; P = 0.2303; Fig. 5C). Lastly, we evaluated overall survival of our targeted biopsy cohort (n = 17) stratified by high or low DCN protein expression by IHC. There was also a trend toward increased overall survival with high DCN expression (Median Survival 26.7 vs. 8.9 months; P = 0.0694; Fig. 5D).

To test the hypothesis that DCN expression may be linked to the VEGF pathway, we compared RNA expression data for the VEGFR 1 & 2 to DCN from the TCGA and IVY GAP databases. There was a positive linear correlation between VEGFR-1 RNA expression and DCN RNA expression in both databases (TCGA: Pearson R^2^ = 0.01996; P = 0.0010; IVY GAP: Pearson R^2^ = 0.6519; P < 0.0001; Fig. 5E,F). Similarly, there was a positive linear correlation between VEGFR-2 RNA expression and DCN RNA expression in both databases (TCGA: Pearson R^2^ = 0.1520; P < 0.0001; IVY GAP: Pearson R^2^ = 0.6912; P < 0.0001; Fig. 5G,H). Additionally, in situ* hybridization* maps from the IVY GAP database found localization of VEGFA in areas histologically annotated as cellular tumor (Fig. 5I), while VEFGR 1 & 2 were localized to areas histologically annotated as microvascular proliferation (Fig. 5J,K), suggesting DCN expression may be specific and localized to areas undergoing microvascular proliferation and related to interaction with VEGFR 1/2.

## Discussion

With the increasingly mounting evidence on the extent of heterogeneity within glioblastoma^117–119^, it is clear that individualization of therapy to a patient’s specific tumor may be the most appropriate treatment strategy. This includes revisiting previously “failed” therapies, such as bevacizumab, to identify patients that may receive a significant benefit from this molecular therapy. We have previously identified diffusion MR imaging phenotypes that can predict patients that will receive a substantial survival benefit from anti-VEGF therapies, including bevacizumab^16–21,116^. While it is clear that this connection exists, the biologic mechanism for this clinical observation has been largely unexplored.

In this study, we establish a connection between increased ADC_L_ measured from DWI and DCN expression, a small proteoglycan belonging to the small leucine-rich proteoglycan family. DCN has previously been shown to soften the ECM via activation of MMPs^33^, as well as by increasing space between collagen based macromolecules^48^. This function of DCN has been utilized to treat fibrosis in the body^34–41^ and central nervous system^42–44^. Given that high ADC is associated with decreased cell^25–30^, fluid^49,50^, and connective tissue density^51–55^, a correlation between high DCN expression and high ADC is reasonable. We found elevated DCN RNA expression and protein expression in tumors with high ADC_L_ and confirmed this association using two large publicly available databases. In addition, we found consistency of this relationship within different areas of an individual tumor, supporting our hypothesis that DCN overexpression may be intimately linked with the high ADC_L_ phenotype.

We sought to explore whether DCN could also serve as a link between ADC imaging phenotypes and favorable response to anti-VEGF therapy. A function of DCN includes inhibition of angiogenesis by high-affinity interaction with VEGFR2 and resultant increased levels of PEG3, a tumor suppressor gene through induction of autophagy^120^. DCN has also been noted to sequester multiple angiogenic growth factors^33^ and suppress tumor cell-mediated angiogenesis through suppression of VEGF mRNA and protein^78^. We localized DCN to areas of microvascular proliferation within glioblastoma tumors, observing DCN in the tunica adventitia of blood vessels within tumor specimens. We identified a negative association between DCN and VEGF-A and a positive association between DCN and VEGFR 1 & 2 and localize VEGFR 1 & 2 to the similar areas of microvascular proliferation in glioblastoma specimens. These associations are in concordance with DCN anti-angiogenic mechanisms in non-glioblastoma tumors, where DCN has been noted to bind to VEGFR^120^ and inhibit production of VEGF^78^. Modulation of the VEGF pathway by DCN may serve as one potential explanation to why patients with increased DCN have increased survival and why patients with increased ADC respond better to anti-VEGF therapies.

This study includes several limitations. First, certain clinical and database analyses suffer from small sample sizes. Furthermore, given the fact that diffusion MRI data acquired on different scanners^121^, bias is introduced for datasets with heterogeneous scanner types. We attempt to address these limitations by combining similar correlative analyses from multiple sources, including databases with large sample size and clinical cohorts with homogeneity in scanner type as well as by examining both transcriptomic and protein expression.

Although we have established a correlative association between DCN and diffusion MR phenotypes, many questions remain regarding the causality of this association. Future studies aimed at further probing this mechanism are warranted, including use of overexpression and knockout studies to examine the ability for DCN expression levels to modulate ADC, inhibit tumor angiogenesis and/or proliferation, and synergize with concurrent anti-VEGF therapy to increase treatment efficacy.

## Conclusions

Increased ADC on DWI of patients with glioblastoma is associated with increased RNA and protein expression of DCN, a proteoglycan that modulates the ECM and has well established anti-angiogenic properties. DCN localizes in glioblastoma specimens to areas of microvascular proliferation, the tunica adventitia of blood vessels within the tumor, and co-localizes to areas of VEGFR. DCN may serve as a link between increased ADC through its modulation of the ECM and favorable response to anti-VEGF therapy through its anti-angiogenic properties.

## Methods

### Patient selection

This study was approved by the “Medical IRB #2” at the University of California Los Angeles in accordance with the Helsinki Declaration of 1964. All patients were provided informed written consent to have advanced imaging and medical information included in our IRB-approved research database according to IRB#14-001261 or IRB#10-000655 approved by Medical IRB #2 at the University of California Los Angeles. Three separate patient cohorts from a single institution were used in the current study: (1) patients who received image-guided biopsies; (2) patients who received resective surgery and had patient-derived orthotopic xenograft (PDOX) avatar models generated from the resected tumor; and (3) patients with recurrent glioblastoma treated with bevacizumab with or without adjuvant chemotherapies. Patients were included if they met the following general inclusion criteria: (1) radiographic evidence of glioblastoma; (2) clinically determined to be eligible for resective surgery or bevacizumab treatment; (3) pre-operative or pre-treatment MR imaging scans including pre- and post-contrast T1-weighted images and diffusion weighted images (DWI or diffusion tensor imaging) of sufficient image quality; (4) measurable contrast enhancing tumor ≥ 1 cm × 1 cm; and (5) ≥ 18 years old.

### Anatomic and diffusion MR imaging

Standards from the international standardized brain tumor imaging protocol^122^ were used to guide acquisition of anatomic and diffusion MR images. These images were obtained on a 1.5 or 3 T scanner (Siemens Healthcare, Erlangen, Germany or GE Medical Systems, Waukesha, Wisconsin. ADC maps were calculated from diffusion sequences via *b* = 0 s/mm^2^ and *b* = 1,000 s/mm^2^ images.

### T1 subtraction maps

Contrast enhanced T1-weighted digital subtraction maps were created in order to isolate the areas of contrast enhancement and negate blood products within tumor regions of interest. The process of generating T1 subtraction maps has been detailed previously^123–125^. Briefly, T1 weighted images with and without contrast were co-registered using affine registration (12 degrees-of-freedom; correlation coefficient cost function, FSL (FLIRT;FMRIB Software Library, Oxford, England). Image intensity was normalized for both images (National Institutes of Mental Health Magnetoencephalography 3Core Facility, 3dNormalize; NIMH MEG Core, Bethesda, MD). Subtraction was performed on a voxel-wise level between normalized and co-registered images. Regions with positive values were segmented to identify regions for ADC analysis (Fig. 1A,B).

### ADC histogram analysis

The methodology for ADC histogram analysis has been described in detail elsewhere^16,18,20,21,126^. Simply, the histograms of ADC values extracted from contrast enhancing regions of interest from T1 subtraction maps were generated. Then, nonlinear regression using a double Gaussian model was performed (MATLAB, Release 2018b Version 9.5.0; The MathWorks Inc., Natick, Massachusetts) via the following equation (Fig. 1C):

$$p(ADC)=f\cdot N\left({\mu }_{AD{C}_{L}}{\sigma }_{AD{C}_{L}}\right)+\left(1-f\right)N({\mu }_{AD{C}_{H}}{\sigma }_{AD{C}_{H}}),$$

p(ADC) = probability of a specific ADC value, *f* = proportion of voxels in the histogram, *N*(μ,σ) = normal Gaussian distribution (mean μ; standard deviation σ), *ADC*_*L*_ = lower Gaussian distribution, *ADC*_*H*_ = higher Gaussian distribution.

### ADC histogram probability maps

In order to evaluate ADC at an individual point of the tumor, surgical biopsy targets were selected using ADC probability maps (Fig. 1D). This is a voxel-by-voxel estimation of the probability the voxel’s ADC measurement was part of the high vs. low Gaussian distribution. The ADC probability density function for the lower Gaussian distribution can be defined as:

$${ADC}_{L}PDF \left({ADC}_{ijk}\right)=({\sigma }_{ADCL} \sqrt{2\pi }^{-1}) \mathrm{exp}\left(\frac{({{ADC}_{ijk}-{ADC}_{L)}}^{2}}{2{\sigma }_{ADCL}^{2}}\right)$$

ADC_L_ PDF = ADC probability density function for lower Gaussian distribution, ADC_ijk_ = ADC measurement at voxel location (i,j,k), ADC_L_ = mean of lower Gaussian distribution, σ_ADCL_ = standard deviation of lower Gaussian distribution.

A parallel equation was used for the ADC probability density function of the higher Gaussian distribution. From this, the estimated likelihood the ADC measurement within a voxel falls within the lower rather than the higher distribution can be described as:

$${I}_{ijk}= \frac{\underset{{ADC}_{ijk}}{\overset{\infty }{\int }}{ADC}_{L}PDF\left({ADC}_{ijk}\right) dADC- \underset{0}{\overset{{ADC}_{ijk}}{\int }}{ADC}_{H}PDF\left({ADC}_{ijk}\right)dADC}{\underset{{ADC}_{ijk}}{\overset{ \infty }{\int }}{ADC}_{L}PDF\left({ADC}_{ijk}\right) dADC+ \underset{0}{\overset{{ADC}_{ijk}}{\int }}{ADC}_{H}PDF\left({ADC}_{ijk}\right)dADC}$$

The calculation yields a value for each voxel (i,j,k) ranging from − 1 to 1 with 1 indicating a 100% likelihood a voxel is represented by the lower Gaussian distribution of ADC values.

### Image-guided biopsy procedures

Pre-operative, post-contrast T1-weighted images and associated ADC probability maps were used to select biopsy targets. The size of each target was 5 mm × 5 mm × 5 mm. Areas of high ADC and low ADC were chosen, taking into account: (1) surgical approach and goal of surgery and (2) avoidance of eloquent cortex and vasculature (Fig. 1E). The three-dimensional coordinates for the various image-guided biopsy targets were fused to preoperative, post-contrast T1-weighted images and uploaded onto a Brainlab Neuronavigation system (Brainlab, Munchen, Germany) prior to surgery (Fig. 1F).

All surgeries were carried out at the University of California Los Angeles between May 2015 and November 2018 by faculty neurosurgeons (L.M.L., R.E.). Brainlab neuronavigation was registered with surface markers in a routine manner^127^. During tumor resection, areas of tumor corresponding to predetermined target sites were confirmed with intraoperative navigation and biopsied (Fig. 1G), while the residual bulk tumor tissue was used for clinical pathologic analysis. There was no change to standard surgical technique and postoperative care in patients that had targeted biopsy specimens obtained.

### Immunohistochemistry (IHC)

Specimen processing and interpretation was carried out by the UCLA Brain Tumor Translational Resource under supervision of a board-certified neuropathologist (W.Y.) Targeted biopsy specimens were formalin-fixed and paraffin-embedded according to standard protocol. Specimens were stained with standard H&E and decorin using anti-decorin antibodies (abcam, ab175404). High resolution images of the stained specimens were obtained and imported into the open source digital pathology image analysis software QuPath^128^. Semiautomatic quantitative measurement of immunostaining was carried out using percent positive pixel values using a preprocessing using QuPath’s “*estimate stain vectors”* for hematoxylin and DAB channels^128^.

### The cancer genome atlas (TCGA), the cancer imaging archive (TCIA), & IVY glioblastoma atlas project (IVY GAP) data

The TCGA Data Portal (https://tcga-data.nci.nih.giv/tcga/) was used to collect gene expression data in the form of level 3 probe collapsed messenger RNA (mRNA) expression data via the Affymetrix HT-HG-U133A Gene Chip. Affymetrix expression data were normalized by robust multichip average (RMA). Genomic subtypes were identified from the available published literature^117^. Clinical data such as overall survival was obtained. MR images including pre- and post-contrast T1-weighted images along with ADC were downloaded from the TCIA (https://cancerimagingarchive.net) for the corresponding TCGA patients. The IVY GAP Data Portal (https://glioblastoma.alleninstitute.org/) was used to collect image data of in situ hybridization tissue, associated H&E stained sections, histological description maps, RNA sequencing data, and survival data. Associated pre-operative, pre- and post-contrast T1-weighted images and ADC images were downloaded.

### Patient derived mouse xenograft studies

A total of 35 patients undergoing resection of new or recurrent GBM underwent pre-operative ADC histogram analysis and after resection, tumor specimen was used to establish patient derived mouse orthotopic xenograft (PDOX) models as described previously^129^. Briefly, a portion of a surgically resected tumor sample (~ 1 g) was mechanically and enzymatically digested followed by sequential MACS MicroBeads (Miltenyi Biotec, Bergisch Gladbach, Germany) depletion of myelin/debris, CD45+ cells, and dead/apoptotic cells. Per protocol, purified tumor cells were then implanted into the brains of 6–8-week-old female nod-scid gamma (NSG) mice to create orthotopic glioma PDOX models^129^. Once tumors engrafted and mice became moribund, whole tumor-bearing brains were harvested and mechanically and enzymatically digested, followed by mouse cell depletion (MACS MicroBeads) to isolate pure tumor cells from xenografted mice. These cells were then used for serial transplantation and/or next generations sequencing consisting of both RNA and exome sequencing.

### Bevacizumab treatment in recurrent glioblastoma

A total of 172 adult patients treated at UCLA for recurrent GBM with bevacizumab with or without chemotherapy were included in this study. Electronic medical charts were queried for clinical characteristics and survival data. Pre-treatment MR images were downloaded for ADC analysis as described above. The average age was 58 years (range 29–83 years), average enhancing tumor volume was 25 cc (range 0.29–144 cc), and average survival from bevacizumab treatment was 9.0 months (range 0.03–40 months). Average ADC_L_ was 1.24 (0.31–2.2) and 81 (47%) patients had high (> 1.24) ADC tumors while 92 (53%) had low (< 1.24) ADC tumors. A total of 17 of these patients underwent targeted biopsies.

### Statistical analysis

All statistical analyses were carried out on GraphPad Prism, Version 4.0c (GraphPad Software, San Diego, California). A P value of < 0.05 was considered statistically significant. Linear correlation was used to evaluate the association of DCN RNA expression with ADC_L_ using the TCGA and IVY GAP data and of DCN IHC positivity with ADC_L_ from targeted biopsies as well as between DCN and VEGFR RNA expression. Unpaired t-tests were used to compare DCN RNA expression in high and low ADC_L_ tumors from bulk RNA sequencing from PDOX models as well as DCN IHC positivity in high and low ADC_L_. Paired t-test was used to compare DCN RNA expression between different histological areas from individual tumors. Kaplan Meier log-rank analysis was used to evaluate differences in survival in patients with different diffusion phenotypes or DCN expression levels.

## Data Availability

Data involved in this study, may be made available on request form the corresponding author [B.E.]. Patient imaging data are not publicly available due privacy restrictions, i.e. presence of personal health information.
